# How are evidence generation partnerships between researchers and policy-makers enacted in practice? A qualitative interview study

**DOI:** 10.1186/s12961-019-0441-2

**Published:** 2019-04-15

**Authors:** Anna Williamson, Hannah Tait, Fadi El Jardali, Luke Wolfenden, Sarah Thackway, Jessica Stewart, Lyndal O’Leary, Julie Dixon

**Affiliations:** 1 The Sax Institute, PO Box K617, Haymarket, NSW 1240 Australia; 2 University of Sydney, Sydney, Australia; 3 University of New South Wales, Sydney, Australia; 4 American University of Beirut, Beirut, Lebanon; 5 University of Newcastle, Callaghan, Australia; 6 Hunter New England Population Health, New Lambton, Australia; 7 New South Wales Health, North Sydney, Australia; 8 Department of Family and Community Services (FACS) Insights, Analysis and Research (FACSIAR), Ashfield, Australia; 9 Western NSW & Far West Local Health Districts, Dubbo, Australia; 10 South Eastern Sydney Local Health District (SESLHD), Carringbah, Australia

**Keywords:** Partnership, policy, co-production

## Abstract

**Background:**

Evidence generation partnerships between researchers and policy-makers are a potential method for producing more relevant research with greater potential to impact on policy and practice. Little is known about how such partnerships are enacted in practice, however, or how to increase their effectiveness. We aimed to determine why researchers and policy-makers choose to work together, how they work together, which partnership models are most common, and what the key (1) relationship-based and (2) practical components of successful research partnerships are.

**Methods:**

Semi-structured qualitative interviews were conducted with 18 key informants largely based in New South Wales, Australia, who were (1) researchers experienced in working in partnership with policy in health or health-related areas or (2) policy and programme developers and health system decision-makers experienced in working in partnership with researchers. Data was analysed thematically by two researchers.

**Results:**

Researcher-initiated and policy agency-initiated evidence generation partnerships were common. While policy-initiated partnerships were thought to be the most likely to result in impact, researcher-initiated projects were considered important in advancing the science and were favoured by researchers due to greater perceived opportunities to achieve key academic career metrics. Participants acknowledged that levels of collaboration varied widely in research/policy partnerships from minimal to co-production. Co-production was considered a worthy goal by all, conferring a range of benefits, but one that was difficult to achieve in practice. Some participants asserted that the increased time and resources required for effective co-production meant it was best suited to evaluation and implementation projects where the tacit, experiential knowledge of policy-makers provided critical nuance to underpin study design, implementation and analysis. Partnerships that were mutually considered to have produced the desired outcomes were seen to be underpinned by a range of both relationship-based (such as shared aims and goals and trust) and practical factors (such as sound governance and processes).

**Conclusions:**

Our findings highlight the important role of policy-makers in New South Wales in ensuring the relevance of research. There is still much to understand about how to initiate and sustain successful research/policy partnerships, particularly at the highly collaborative end.

**Electronic supplementary material:**

The online version of this article (10.1186/s12961-019-0441-2) contains supplementary material, which is available to authorized users.

## Background

Bridging the evidence–practice gap in health services has the potential to make a substantial contribution to improving health outcomes and service efficiencies [1, 2] and to reducing research waste [1]. Proposed solutions to bridging this gap originally focussed heavily on strategies to enhance the dissemination of research findings to research users [2, 3] or on building the capacity of policy-makers and clinicians to engage with and use research evidence [4]. More recently, focus has turned to the potential for evidence generation partnerships between researchers and knowledge users, such as policy-makers, community groups, clinicians and consumers, to improve the availability of relevant, timely evidence to help inform decision-making [5, 6]. There has been a proliferation of research funding initiatives internationally that require researcher/knowledge user collaboration [7] and increasing demands on researchers to demonstrate that their work has had an ‘impact’ on policy or practice [8–10].

A number of research approaches that centre around evidence generation partnerships have been outlined in recent years, including integrated knowledge translation [5, 11], participatory action research [12, 13] and engaged scholarship [14, 15]. Most of these approaches emphasise the co-production of knowledge [6], whereby researchers and those most likely to use or be affected by the evidence produced, work together to produce evidence. By combining the varied skills and expertise of these groups, it is hypothesised that the resultant research will have greater relevance to knowledge users and will more likely be used in decision-making [16, 17].

Much has been published about many of these approaches from a conceptual standpoint [18, 19], and there has similarly been a considerable amount of literature examining how evidence generation partnerships function [13, 20, 21]. Most of this research, however, does not explore evidence generation partnerships specifically between researchers and policy-makers [22, 23], focusing instead on partnerships with community groups [13], clinicians [24] and others. All of these partnership combinations are likely to share some common features such as a requirement to take into account the diverse perspectives and needs of all partners; however, the policy-maker/researcher combination is likely to present some unique partnership challenges and opportunities due to the vastly different work environment and pressures that these groups face [25–27]. Further, while rarely the case with most other common partnership combinations, evidence generation partnerships between policy agencies and researchers may be initiated and funded by the policy agency themselves or be funded through external research grants (such projects may be more likely to be researcher-initiated). These different modes of funding and initiation may have important implications for how a partnership functions [28]; however, this has rarely been explored. Thus, there remains much to be learnt about how research/policy partnerships are enacted in practice [29] and how best to bring the diffuse skills, needs and priorities of researchers together effectively [30, 31].

The current paper is the first step in a broader initiative which aims to develop a set of tools to help facilitate successful research/policy partnerships around evidence generation activities. In this paper, we explore the views and experiences of researchers and policy-makers with extensive expertise in research partnerships regarding why and how researchers and research users work together in New South Wales, Australia, and the factors which underpin successful research partnerships. In particular, we address the following research questions:

1. Why do researchers and policy-makers choose to work together? What are the perceived benefits?
2. How do they work together? Which partnership models are most common?
3. What are the key (1) relationship-based and (2) practical components of successful research partnerships?

## Methods

### Participants and setting

Data was collected between October 2017 and April 2018. We sought to interview participants from two groups; namely (1) researchers experienced in working in partnership with policy in health or health-related areas and (2) policy and programme developers and health system decision-makers (hereafter policy-makers, defined as “*someone employed in a policy agency who drafts or writes health policy documents or develops health programs, or who makes or contributes significantly to policy decisions about health services, programs or resourcing*” [32]) experienced in working in partnership with researchers. Participants were purposively identified by an Advisory Committee guiding this project. The role of the Advisory Committee was to provide expert advice on issues relating to the scope, implementation and dissemination of a programme of work centred on partnership research of which the current paper is a part. The Advisory Committee included an approximately equal mix of researchers and senior policy-makers, selected for their expertise in partnership research, and their extensive knowledge of researchers and policy-makers who work in partnered research. Advisory Committee members asked the permission of potential participants before passing on their contact details to the study team.

Participants were eligible to participate in the current study if they were nominated by a member of the Advisory Committee based on the criteria that they (1) were a researcher or policy-maker; (2) worked primarily in health or health-related areas (e.g. social care); (3) had extensive experience in partnering with researchers (for policy-makers) or policy-makers (for researchers) on research projects (partnership on at least five projects); and (4) were employed by a university, policy or programme agency or Local Health District. All participants provided written, informed consent to participate.

### Data collection

A semi-structured approach was used to elicit participants’ opinions and experiences regarding the barriers and facilitators to successful evidence generation partnerships amongst researchers and research users in accordance with best practice guidelines (Additional file 1: Appendix 1) [33]. Interviews were conducted by AW, a researcher with extensive expertise in conducting research in partnership with policy-makers. Each interview sought information on why the interviewee engaged in partnership research and the perceived risks and benefits, how the interviewee would characterise the types of partnership models they have engaged in (and the advantages and disadvantages of each), characteristics of successful and unsuccessful partnerships (and the factors associated with each), and indicators and predictors of impact. Interviews were conducted in person or by phone depending on the preference of the interviewee and ranged from half to one hour in length. Data was audio recorded before being transcribed by a person with no personal or professional connection to the participants involved in the study.

### Analysis

Data was analysed thematically. Two researchers (AW and HT) independently read all of the transcripts and coded the data to discern themes inductively. No predetermined framework was used to guide analysis. The researchers met regularly to review the draft codes and themes throughout the process until agreement was reached regarding the final version. Synthesised data and emerging themes were reviewed by the Advisory Committee who participated in sense making of emerging data.

## Results

A total of 18 key informants participated in interviews, of whom 7 were primarily researchers and 11 were currently primarily policy-makers. All participating researchers were employed by universities at the Associate Professor or Professor level and had more than 15 years research experience in public health research. All of the policy-makers who participated were employed at a Manager level or above by government agencies whose work focussed on health or health-related issues (such as social care). Six of the policy-makers had PhDs and/or had previously been employed as researchers and were thus able to bring both a researcher and a policy-maker lens to their analysis of research partnerships. All participants, except for one researcher, were based in New South Wales, Australia. The themes which emerged from the interview with the participant from outside New South Wales were consistent with those which emerged more broadly.

### Why do researchers and policy-makers choose to work together?

Three major themes emerged regarding why researchers choose to work with policy, namely (1) increasing the likelihood of research impact; (2) gaining access to sought after resources; and (3) obtaining funding. The reason most commonly cited by researchers for wanting to partner with policy-makers to conducting research was the belief that the resultant research would more likely have an impact on policy and thus contribute to improving health.

“*I do research so that I can improve health… the main way in which research impacts health is through policy... Research without any sort of policy engagement will often sit on the shelf and do nothing…*” Researcher

Many researchers were also motivated to partner with policy-makers due to the access to otherwise unavailable resources this could facilitate (such as routinely collected data held by agencies or the data required to evaluate large-scale government policies or programmes). Researchers also reported seeking collaborations with policy-makers due to the funds attached to the work, either through completing a tendered project or, preferably, receiving funds to carry out a project the researchers had initiated or co-produced in collaboration with a relevant agency.

Five major themes emerged regarding what policy-makers perceived they gained by partnering with researchers, namely (1) access to additional skills; (2) links to researchers who can be called on for timely, informal advice; (3) access to the networks of their research collaborators; (4) the creation of high quality, relevant evidence; and (5) public, evidence-based support for government decisions. Policy-makers most commonly reported seeking to collaborate with researchers in order to gain access to skills and capacity over and above that already available within their agency.

“*…the reason you go to a researcher is that they’ve got the expertise or the resources or the time or whatever in order to be able to collect that information that you need and therefore that’s helpful to you.*” Policy-maker

Having established collaborations with researchers was also said to create a situation in which policy-makers felt able to contact these researchers when they required fast expert advice on a relevant issue. Policy-makers also noted that their collaborators often assisted them in making links with researchers outside of the collaboration who possessed additional specific skills or knowledge they needed. Policy-makers were also driven to collaborate with researchers in order to create high quality evidence that was seen to have credibility to support their work.

Collaborations with researchers were also seen to be helpful when the researcher elected to support government decisions in the public domain, for example, by speaking about the evidence base underpinning policy, programme or health service delivery decisions. While research users did not report requesting assistance of this nature, they appreciated it when provided.

### How do researchers and policy-makers collaborate on research projects?

Four major themes emerged in relation to researcher/policy-maker collaboration, namely (1) wide variation in extent of collaboration; (2) policy agency-initiated partnerships; (3) researcher-initiated partnerships; and (4) co-produced partnerships. Most participants agreed that “*someone has to have the initial idea*” for a partnership; as such, participants agreed that most partnerships could be categorised as either policy agency initiated, including projects put out for tender by agencies and commissioned work, or researcher initiated, either through a partnered funding application to an external granting body or by a direct approach to an agency to fund a particular piece of work. Once these partnerships began, there was said to be a considerable level of variation in the extent to which the researchers and agency staff involved collaborated from almost no collaboration through to high levels of collaboration throughout all stages of the research process. This sustained, high level collaboration was considered by participants to characterise co-production. Policy-makers and researchers tended to nominate research projects centred around evidence reviews and analysis of existing datasets as requiring minimal collaboration, while closer collaboration was considered important when conducting interventions and evaluations.

“*So, I see partnerships as a sort of continuum. At one end is that transactional type interaction, through to co-production at the other end.*” Policy-maker

However, these partnership categories (policy- or researcher-initiated and extent of collaboration) were not seen to be entirely clear cut, with some researchers and policy-makers explaining that, as some policy agencies move towards embedding researchers within their teams and sometimes funding research centres, the distinction between policy-makers and researchers can sometimes be blurry.

A few policy-makers felt that highly collaborative, co-produced research partnerships were always preferable; however, most participants reported that various levels of collaboration could be effective depending on the situation.

“*I guess again we don’t come at it from which model works best. It’s more like what are the requirements of the project*.” Policy-maker

#### Policy agency-initiated partnerships

A range of shared benefits (two subthemes), perceived unique risks to researchers (five subthemes), perceived unique benefits to researchers (two subthemes), and unique risks to policy-makers (five subthemes) of policy agency-initiated partnerships were identified (see Table 1 for illustrative quotes). Most researchers and policy-makers alike agreed that policy-makers are best placed to identify the critical issues around what evidence is needed to guide their decision-making. Thus, agency-initiated work was thought by both researchers and policy-makers to often be particularly well targeted for real-world impact.

**Table 1:** Illustrative quotes regarding policy agency-initiated partnerships

Shared benefit	Benefit to researchers	Risk to researchers	Risk to policy agencies
Agency best placed to identify critical real-world issues “*I feel like the agency’s good at identifying the problem and that’s often unknown to researcher. They’re just not in the trenches. They just kind of can’t see it.*” Researcher	Stepping stone to future collaborations “*…it’s a long game, these relationships. Doing the work that they’re commissioning but also with a view to potentially collaborating with them on bigger things that we might potentially have an interest in, in the future.*” Researcher	Opportunity cost “*I have a research team of people who are ambitious… In an ideal world they’ll be doing projects which are targeting high impact, international journals. The sort of work that we do for government here isn’t that sort of work. So, it does come at that cost as well.*” Researcher	Researchers not engaging with complexity “*Sometimes they get very narrow in terms of what they get interested in or they need to define things in a very narrow way that means that they can measure it, so it makes it easier for them, but it doesn’t necessarily answer the questions that you want. When from our point of view we’ll think, they’re just not even trying to get how complex, they just want it to be simple because that’s their area of expertise.*” Research user
Evidence generated more likely to be used in policy or practice “*I think if they’ve got a specific evidence need and know the evidence they need to gather or synthesise to answer it, I think that’s quite an acceptable process that will probably lead to really quick translation because they need the evidence and as soon as they get it, they’ll make the decision on its basis.*” Researcher		Changing political environment “*So honestly my best work was dead in the water within six months because of politics.*” Researcher	

Small, short term, policy-initiated projects were rarely seen by researchers as offering opportunities to conduct ‘cutting edge research’, and reportedly often did not generate publications or turn a profit. Researchers reported willingness to engage in these and other types of agency-initiated projects anyway as they were seen as an effective way to build a relationship with a policy agency and gain a better understanding of the policy context in the hopes of developing more advantageous collaborations.

On the other hand, most researchers outlined a number of potential risks or costs that were sometimes associated with agency-initiated work. Chief among these was a perceived opportunity cost, with time spent working on agency-initiated research sometimes resulting in less traditionally valued research outputs (such as peer-reviewed publications) than time spent on researcher-initiated work. Indeed, policy-makers and researchers alike reported that, as policy-makers often needed evidence around a specific issue, within a specific timeframe and at a specific cost, research methods needed to be determined pragmatically, rather than striving for the most scientifically excellent design. In addition, the evidence produced was sometimes rendered irrelevant by changes in the political environment.

Time was again a concern amongst researchers when the amount of time spent to complete a project to an agency’s satisfaction was seen to ‘blow out’. Researchers and some policy-makers reported that this was often due to agencies not being “*clear* [about] *what they want*”, resulting in a more iterative process than was anticipated. This challenge was also sometimes seen to be related to agencies not disclosing key information to researchers, limiting their understanding of the context and what was required.

Policy-makers also reported potential risks related to engaging with researchers in agency-initiated research, including not receiving the information or evidence they required, or it being of poor quality, or delivered well after the agreed deadline. Another commonly reported barrier was researchers overpromising to win a tender but not being able to deliver on these promises. Frustrations were sometimes said to arise due to researchers ignoring the complexity of the issue being investigated.

Finally, policy-makers reported that, in policy-initiated projects, researchers often failed to adequately synthesise the reported evidence and/or provide recommendations for action. As recommendations for action were reportedly often one of the key outcomes sought by agencies in these partnerships, this was particularly disappointing.

#### Researcher-initiated partnerships

One shared benefit related to research-initiated partnerships was commonly identified by researchers and policy-makers. Policy-makers also identified a range of risks associated with such partnerships (four subthemes). Researchers considered that partnerships they led conferred a range of important benefits (three subthemes), but also some risks (two subthemes) (see Table 2 for illustrative quotes). Researcher-initiated partnerships were seen by policy-makers as an important source of innovation, but not necessarily of major immediate relevance to their work. Despite this, if the policy-makers agreed to partner on a researcher-initiated project, they reported that they did expect to engender benefits from this. Nonetheless, one of the key risks noted for researcher-initiated partnerships was that the evidence produced did not fill an evidence need for the agency, or that it took so long to produce that, by the time it was available, it was no longer relevant.

**Table 2:** Illustrative quotes about researcher-initiated partnerships

Shared benefit	Benefit for researchers	Risk for research user agencies
Innovation “*Investigator driven research is fine, we actually encourage it all the time. That’s where ideas come up, where things that we haven’t thought of - we don’t have the capacity to think of everything and have every partner and be at every meeting. So certainly, there’s a very important role for investigator driven research.*” Research user	Increased opportunity for cutting edge research “*…if the researcher is initiating it…it’s more likely to be characterised by sort of something quite new in terms of maybe methods or thinking outside the square.*” Policy-maker	Long timelines “*…they* [researcher-initiated partnership projects] *can take a long time, longer than you need or have available. So you don’t get the answer you want when you want it, you get it eventually, but it may be too late by that point, in which case it’s something of a waste of time.*” Policy-maker
		No acknowledgement “*...they* [policy-makers] *weren’t acknowledged. They weren’t told that a peer review paper had been produced, it just came out…There wasn’t* [1] *the respect to even ask to produce the paper, and* [2] *to even ask whether someone could be an author on it. Someone who put a lot of intellectual property into the project.*” Policy-maker
		Lack of practical outcomes “[A risk is that researchers were not]*…making recommendations in reports that speak to those issues of policy and practice implications, rather than being airy fairy high falutin, more research needs to be in A, B, C. It’s not very helpful.*” Research user

This risk was seen to be exacerbated by another commonly noted risk of this partnership type, namely that the researchers take over and offer little opportunity for policy-makers to help shape the research agenda. Relatedly, many policy-makers reported previous dissatisfactions related to researchers not sharing credit for the work the partnership produced, for example, through co-authorship or shared publicity.

Researchers cited significant benefits associated with researcher-initiated partnerships, including the opportunity to focus on their specific research interests and employ more ambitious study designs, increasing their chances of obtaining peer-reviewed grants and high impact journal articles. The chief risks researchers reported in researcher-initiated research partnerships were that the policy-maker partners did not engage (attend meetings, provide information and advice) or did not deliver on promised resources such as access to particular datasets.

#### Co-produced partnerships

All participants reported that co-production was a worthy goal and likely to be highly effective when done well (four subthemes). Some regarded it as the most effective partnership model for real world impact (see Table 3 for illustrative quotes). Achieving co-production was also noted to be difficult, with common challenges (two subthemes) and a range of facilitators (four subthemes) identified.

**Table 3:** Illustrative quotes regarding co-production

Shared benefit	Shared challenge
Research more likely to be used “*…what increases the likelihood of research being used is if it’s coproduced, because then we’re actually interacting all the time. We’re talking, we know what the priorities are, and we’re actually setting an agenda for research and we’re collaborating on that…if we’re involved in the process from day dot, involved in its implementation, of course we’re going to use it, more likely to use it aren’t we?*” Policy-maker	Co-production difficult to achieve “*I think the agency-led or researcher-led in my experience is common. But we have pursued that co-led idea, because I think that could have so many benefits. We haven’t - I don’t think we’ve had a lot of success with it, but I think it’s a really great model.*” Policy-maker
Merging of experiential and scientific knowledge “*…there is a lot of evidence that is really more implicit, part of the tacit knowledge, part of the* [experiential] *knowledge that stakeholders have, and that engagement process and coproducing evidence of what works and what does not work is really quite promising. I tell you that in many cases, even systematic reviews…sometimes you might have strong evidence, but it doesn’t mean that at the level of implementation in governing contract it would work. This is where the knowledge coproduction piece is important.*” Researcher	Co-production may require shared risk “*… you do need shared risk. Whether it’s financial shared risk, outcome shared risk, reputational shared risk, and that’s what kind of drives a lot of things, and sort of shared input in some ways, whether it’s in kind, or not in kind, or whatever it is. So, I think shared input, and shared risk, I think, is something that, probably, people might not want to say, but it’s very true, because that’s where you’ll get a lot of input*.” Policy-maker
Keeps research relevant “*I think it makes the research better….you can check in with the people who are going to be most impacted by the research as you go along. Make sure you don’t go off on an academic tangent and lose relevance. Because real-world questions are messy and crowded, and often not exactly as they would be in the perfect academically articulated research questions.*” Researcher	

The benefits of co-production were seen to derive in part from the greater levels of engagement between all members of the team seen to be implicit in this model, driven by mutual aims and interests. This high level of engagement was in turn seen to allow the complementary skills and knowledge of all partners to be optimised. Resulting from this, a core strength of co-production was thought to be its ability to integrate tacit, experiential knowledge with traditional ‘evidence’, resulting in particularly nuanced and relevant outputs. Co-production was also seen to provide opportunities for frequent ‘relevance checks’ to ensure that the research being undertaken remains in line with ‘real world’ priorities.

Nonetheless, while co-production was the goal for many participants, some noted that, thus far, they had not been able to achieve it, with either the policy or the researcher partners dominating partnership projects in practice. Multiple reasons were given for the reported tendency for one group to dominate research partnerships, ranging from lower expectation amongst one group that the project might benefit themselves or their agency resulting in reduced engagement, to the dominating group offering little real opportunity for their partners to contribute to shaping the research agenda. The most frequently mentioned facilitators of co-production were things that allowed long-term relationships and trust to develop between researchers and policy-makers, namely stability of staff at policy agencies and policy agency-funded research centres.

### What does ‘success’ in research partnerships look like?

Seven subthemes were identified in relation to the characteristics of successful partnerships. Key informants most commonly described a successful partnership as one where “*all stakeholders are happy with the outcomes*”. Expanding further, common perceptions of successful partnerships included all parties having a shared understanding of why they were partnering and what this would involve, the planned deliverables being delivered to a high standard and relevant, usable evidence being generated. Many participants noted that a successful partnership produces more than any partner could have alone and that it results in mutual gain. Creating evidence that impacts on policy and practice was considered an indicator of success, but as participants recognised that impact is influenced by a complex array of factors, they considered that partnerships could be considered successful even in the absence of this.

### What are the components of successful research partnerships?

Participants identified a range of relationship-based (six subthemes) and practical facilitators (six subthemes) of successful partnerships as well as three cross-cutting facilitators (Table 4).

**Table 4:** Major themes regarding relationship-based and practical facilitators of successful partnerships

Relationship-based components	Practical components
Shared aims and goals	Taking time at the outset to ensure there is a shared understanding of the project
Understanding each other’s needs and drivers	Agreed governance structures and processes (including publication policy)
Mutual respect	Adequate funding
Open communication	Clearly specified timelines and deliverables
Taking time to build a relationship OR a pre-existing relationship	All delivering as promised
Trust	Ongoing engagement
Cross-cutting facilitators of successful partnerships	
Flexibility	
Sharing wins and credit	
Mutual benefit	

Shared aims and goals were seen as the fundamental building block of successful partnerships, and something that motivated partners to withstand the difficulties and challenges that can emerge over the course of partnerships.

“*So that’s my little tip there. All the energy at the start has to be about where you want to finish and why a partnership is important, because it will also help you during the times when the partnership’s tested, to remind yourself why working together was important in the first place.*” Researcher

Participants were also keenly aware of the differences between researchers and policy-makers in relation to their needs and goals. For example, researchers are generally expected to demonstrate their productivity through publications and funded grants, whereas policy-makers need to provide advice or develop policies or programmes around complex issues, often with very little preparation time. Both groups emphasised the necessity of understanding and attempting to accommodate these different needs.

“*… it’s the* r*elationship building that’s critical, being prepared to put the time and effort into the relationship building and having the ability to empathise with the perspective of someone who’s trying to run a health service or someone who’s having to make a policy decision…*” Researcher

### Practical components of successful partnerships

All of the practical components of successful partnerships were seen to be underpinned by the need to take time at the outset of the partnership (and throughout) to ensure that ‘everyone is on the same page’. Practical ways of doing this that were frequently cited included documenting and agreeing on timelines, deliverables and who is responsible for what at the outset (but allowing some flexibility for when changes arise), agreeing on governance structures and processes and a publication policy (which specifies the potential role of research users in publications). Adequate funding was seen as another core practical component of success, as was all parties delivering as promised. Ongoing engagement, expressed through attendance at meetings, participation in discussions and provision of feedback, for example, was also seen as key. Mutual benefit was cited by all participants as fundamental to partnership success.

“*The best partnerships are clearly win/win…Where it becomes one-sided, all the gains being on one side at the expense of the other, that’s where things fall apart because the side that’s not gaining is thinking well, what am I doing here?*” Researcher

## Discussion

Our findings suggest that both researcher-initiated and policy agency-initiated evidence generation partnerships were common among researchers and policy-makers in New South Wales. While policy-initiated partnerships were thought to be the most likely to result in impact [34], researcher-initiated projects were seen to play an important role in advancing the science and often provided researchers with more opportunities to achieve the outputs valued in their profession such as high impact publications. In keeping with the literature, participants acknowledged that levels of collaboration varied widely between research/policy partnerships [16, 20, 28, 35, 36].

Co-production, or collaboration across all stages of the research process, was seen by some participants as the ideal model for collaboration, increasing the relevance of the research produced and the likelihood of impact [37–39]. Others considered that this time and resource-intensive way of working was not always warranted [40]. While agreeing aims, goals and deliverables collaboratively was always viewed as essential [41], these informants suggested that, once this was accomplished, some types of projects, such as analyses of large datasets and evidence reviews, could be completed largely independently by researchers with no loss of quality or relevance. In contrast, co-production was seen to be particularly worthwhile in relation to evaluation and implementation projects where the tacit, experiential knowledge of policy-makers provided critical nuance to underpin study design, implementation and analysis. Of note, several policy-makers suggested that, while ideally they would like to co-produce research, in practice they had not found this to be possible. Participants suggested that trusting, long-term relationships were generally a precursor to co-production. These were seen to be facilitated by policy agencies having a stable workforce; frequent staff changes in policy agencies are frequently cited as a barrier to successful evidence generation partnerships in the literature [31, 42] and policy agency-funded research centres [43].

Key informants in the current study highlighted a clear distinction between researcher and policy agency-initiated partnerships. In keeping with the literature, policy-makers were thought by both groups to be best placed to identify what evidence is needed to guide real world decision-making [5, 44, 45]. The relevance of the ensuing research and the ability of policy agencies to utilise it meant that policy agency-initiated work was reported to be particularly likely to lead to real-world impact [22, 34]. Achieving such impact was the primary motivation reported by researchers for engaging in evidence generation partnerships [22]. Potential opportunity costs were also noted, including time taken away from producing ‘world class’ research [28, 46], challenges agreeing on final outputs [28, 29], unexpectedly iterative processes [47] and the potential for the evidence produced to remain unused due to changes in the policy landscape [29].

Many of the key frustrations expressed by policy-makers around policy agency-initiated partnerships in the current study have been less commonly reported in the literature. These include researchers overstating what is possible in order to win tenders (leading to disappointment with the final product), choosing only to focus on the elements of a topic that interest them, or oversimplifying things in order to make them ‘doable’ and failing to fully engage with the complexity of an issue. As systems thinking approaches gain momentum in research [48, 49], it will be interesting to observe whether this last challenge becomes less common. A key frustration noted by policy-makers was the failure of researchers to provide recommendations for action, one of the main outcomes reportedly sought by agencies when they initiated partnerships with researchers [28, 50, 51]. Despite the noted risks, researchers stated that even small agency-initiated projects were worthwhile as relationship-building and learning exercises [27, 52], which may later help to underpin partnerships they felt to be more favourable to themselves. Policy-makers for their part continued to initiate evidence generation partnerships in order to access additional capacity and expertise to produce high quality, credible evidence to inform their work.

Researchers in the current study placed particular value on researcher-initiated partnerships. These were considered to offer the opportunity to conduct research that was both ‘scientifically excellent’, and likely to have impact. Policy-makers, on the other hand, noted that, while sometimes providing useful evidence, these projects were less often of immediate relevance or importance to them. Unsurprisingly then, the major risk researchers reported in researcher-initiated partnerships was that policy-makers did not engage [29, 53]. This suggests that even researchers who aim to conduct research with real world impact, and who are experienced in working partnership with policy, are often not in alignment with policy agencies in their opinions as to research priorities. This finding is consistent with the large body of literature describing the widespread differences between the research and policy worlds [26, 27, 37, 54], whereby researchers most often attempt to ‘push’ their research to policy agencies rather than ascertaining when there may be a policy ‘pull’ for evidence and building at least some of their research agenda to align with this [55, 56]. One mechanism governments in New South Wales [57] and others internationally [58–61] employ to help develop long-term collaborations with researchers and increase the production of policy-relevant research is government-funded research centres. While as yet there have been few evaluations of such initiatives, it seems a potentially promising method of facilitating productive, lasting partnerships.

Many of the key barriers and facilitators reported to underpin partnership success or failure noted in the current study are consistent with those reported in the literature. Many of these appeared to speak primarily of a clear need to ensure that the different needs and drivers of all parties were understood, valued and accommodated as much as possible. Thus, participants underlined the foundational need for shared aims and goals, ensuring that all parties were benefiting from the collaboration and maintaining open communication, trust and mutual respect [16, 20, 21, 27, 42].

Amongst our key informants, practical tools were also thought to be of great value in supporting a well-functioning partnership; these included having clearly documented and agreed timelines, deliverables, workplans, publication policies and governance structures [31, 53]. While some groups appear to have well-developed methods in this area, this appears to be a partnership capacity gap for others. As noted elsewhere [29, 31], ongoing engagement, expressed through things such as attendance at meetings, participation in discussions and provision of feedback and presenting findings as they emerge, was described as critical to keeping the project on track and maintaining relationships.

A strength of the current study is the participation of both researchers and policy-makers who are highly experienced in working in partnership on evidence generation activities as we expected this group to be able to provide considerable insight into the complexities of research/policy partnerships. By exploring their views broadly, rather than in relation to a particular project, we were able to draw out their key learnings from working in partnership on evidence generation activities. We acknowledge the limitations inherent in the small sample size utilised here, the fact that most of our participants were from New South Wales and that our sample was not randomly selected. Indeed, the experiences of these highly practiced individuals may diverge from those of researchers and policy-makers who have less experience of participating in research/policy partnerships, or who work in settings where such partnerships are less common. Although many of our findings align with the existing literature, these limitations mean that our findings may not be broadly generalisable.

There is still much to understand about how to initiate and sustain successful research/policy partnerships, particularly at the highly collaborative co-production end. Our findings highlight the important role of policy-makers in ensuring the relevance of research, mostly in evaluation and implementation projects where co-production appears to be particularly valuable. The next phase of our work will seek to provide a more detailed exploration of the specific practical strategies, including governance approaches, that can be put in place to facilitate strong and effective evidence generation partnerships between researchers and policy-makers.

## Additional file

Additional file 1: **Appendix 1.** Partnership research key informant interviews: policy, programme or health service deliverers. (DOCX 32 kb)
