# Supplementary material for: How are evidence generation partnerships between researchers and policy-makers enacted in practice? A qualitative interview study
Source: Health Res Policy Syst. 2019 Apr 15;17:41. doi: 10.1186/s12961-019-0441-2 (PMC6466802; doi:10.1186/s12961-019-0441-2)
Supplement: Supplementary file 1 — Appendix 1. Partnership research key informant interviews: policy, programme or health service deliverers. (DOCX 32 kb) [file 12961_2019_441_MOESM1_ESM.docx]

# Additional file 1: Appendix 1.

## Partnership research key informant interviews: policy, program or health service deliverers

**Interview guide**

Thank you for speaking with us today about your experiences in partnering with researchers on evidence generation projects – these might include anything from evaluation and data analyses to rapid reviews of evidence to structured engagement with stakeholders. You have been invited to participate due to your extensive experience in partnership projects. We are hoping to learn about how partnership projects work, including any common success and failure factors, and what they actually deliver.

1. Can you tell me a little bit about your experience in working in partnership with researchers on evidence generation projects in general? About how long have you been involved in this type of work? What types of partnerships have you been a part of?
2. Why do you partner with researchers? What do you see as some of the potential benefits? What are the risks?
3. What do you think the potential risks and benefits are for researchers?
4. How do you choose which researchers to partner with?

*I would now like to ask you a bit about the types of partnerships you have been involved in and how they have differed.*

1. People sometimes distinguish between partnerships that are researcher led versus led by a policy or health service organisation versus co-produced (or equally led by a mix or researchers and policy, program or service deliverers throughout the entire research process). Does that distinction ring true to you? If not, is there a different way you might categorise the types of partnerships you have been involved in?

Thinking about the different partnership models you have described, is there one you would consider to be the ‘best’ in general? Why? What needs to happen to start a project using that model and then to make it work.

1. Are there any models that you have found to be particularly unlikely to work? Why?

*I would now like to ask you a bit about what makes an evidence generation partnership successful from your point of view?*

1. Firstly, how would you characterise a successful partnership?
2. In your experience, what types of things do you think have led to a partnership project being successful? Does it differ depending on type of project?
3. What are some common barriers to partnership success you have come across?

*Now I’d like to ask you a bit about partnerships that don’t work so well.*

1. From your point of view, what are some common indicators that a partnership has not worked?
2. What kinds of things most commonly drive suboptimal partnerships?

*Now I would like to talk a bit about impact. In general, what do you consider to be indicators that research has had an impact (within your agency and beyond)?*

1. What factors do you think increase the impact of evidence in your setting?
2. (if not already mentioned in response to the previous question) In your experience, what partnership models have resulted in the greatest impact? What factors do you think drove that?

Thank you for your time.
